# Diagnostic and therapeutic use of oral micronized progesterone in endocrinology

**DOI:** 10.1007/s11154-024-09882-0

**Published:** 2024-04-23

**Authors:** Eleni Memi, Polina Pavli, Maria Papagianni, Nikolaos Vrachnis, George Mastorakos

**Affiliations:** 1 Unit of Endocrinology, Diabetes mellitus, and Metabolism, Aretaieion Hospital, School of Medicine, National and Kapodistrian University of Athens, Vas. Sophias Av. 76, 11528 Athens, Greece; 2 Department of Nutrition and Dietetics, School of Physical Education, Sport Science and Dietetics, University of Thessaly, 42100 Trikala, Greece; 3 Endocrine Unit, 3rd Department of Pediatrics, Hippokration Hospital of Thessaloniki, Aristotle University of Thessaloniki, 54642 Thessaloniki, Greece; 4 Third Department of Obstetrics and Gynecology, Attikon General Hospital, School of Medicine, National and Kapodistrian University of Athens, Rimini Str. 1, 12462 Chaidari, Athens, Greece; 5 St George’s NHS Foundation Trust Teaching Hospitals, St George’s University of London, London, UK

**Keywords:** Micronized progesterone, Amenorrhea, Oligomenorrhea, Menopause, Perimenopause, Hormone therapy

## Abstract

Progesterone is a natural steroid hormone, while progestins are synthetic molecules. In the female reproductive system, *progesterone* contributes to the control of *luteinizing hormone* and *follicle-stimulating hormone* secretion and their pulsatility, *via* its *receptors* on the *kisspeptin*, *neurokinin B*, and *dynorphin* neurons in the hypothalamus. *Progesterone* together with *estradiol* controls the cyclic changes of proliferation and decidualization of the endometrium; exerts anti-mitogenic actions on endometrial epithelial cells; regulates normal menstrual bleeding; contributes to fertilization and pregnancy maintenance; participates in the onset of labor. In addition, it exerts numerous effects on other endocrine systems. *Micronized progesterone* (*MP*) is *natural progesterone* with increased bioavailability, due to its pharmacotechnical micronized structure, which makes it an attractive diagnostic and therapeutic tool. This critical literature review aims to summarize and put forward the potential diagnostic and therapeutic uses of *MP* in the field of endocrinology. During reproductive life, *MP* is used for diagnostic purposes in the evaluation of primary or secondary amenorrhea as a *challenge test*. Moreover, it can be prescribed to women presenting with amenorrhea or oligomenorrhea for induction of withdrawal bleeding, in order to time blood-sampling for diagnostic purposes in early follicular phase. Therapeutically, *MP*, alone or combined with *estrogens*, is a useful tool in various endocrine disorders including primary amenorrhea, abnormal uterine bleeding due to disordered ovulation, luteal phase deficiency, premenstrual syndrome, polycystic ovary syndrome, secondary amenorrhea [functional hypothalamic amenorrhea, premature ovarian insufficiency], perimenopause and menopause. When administrated *per os*, acting as a *neurosteroid* directly or through its metabolites, it exerts beneficial effects on brain function such as alleviation of symptoms of anxiety and depression, asw well as of sleep problems, while it improves working memory in peri- and menopausal women. *Micronized progesterone* preserves full potential of progesterone activity, without presenting many of the side-effects of *progestins*. Although it has been associated with more frequent drowsiness and dizziness, it can be well tolerated with nocturnal administration. Because of its better safety profile, especially with regard to metabolic ailments, breast cancer risk and veno-thromboembolism risk, *MP* is the preferred option for individuals with an increased risk of cardiovascular and metabolic diseases and of all-cause mortality.

## Introduction

*Progesterone* is a natural steroid hormone, while *progestins* are synthetic molecules. In 1929, *crystalline progesterone* was isolated from the corpus luteum of sow. In 1934, its chemical structure was determined by Adolf Butenandt (Nobel Prize 1935). *Progestins* appeared in the late 30s and were initially used for birth control in the 50s. They were a more effective treatment than *per os* administrated *natural progesterone* which presented poor absorption and rapid metabolic decay. In 1984, *micronized progesterone* (*MP*), *natural progesterone* with increased bioavailability was synthesized. Its pharmacotechnical *micronized* structure preserves its potential of activity without presenting many of the side-effects of *progestins* [1]. Micronization, first employed in France, is currently employed in other European countries and in USA as well.

Because *MP* appears as an attractive diagnostic tool and a therapeutic alternative to *progestins*, this critical literature review aims to summarize and to put forward the potential diagnostic and therapeutic uses of *MP* in the field of endocrinology. To identify publications regarding the role of *MP* in endocrinology and its advantages, a systematic literature search for human studies was conducted in three electronic databases (PubMed, Cochrane and Medline) until september 2023 using combinations of the key terms “micronized progesterone”, “bioavailability” and “advantages in endocrinology”. In addition, a manual search of key journals and abstracts from the major annual meetings in the fields of endocrinology and reproductive gynecology was conducted.

## Biochemistry and physiology of progesterone

### Biochemistry of progesterone

**Fig. 1:**
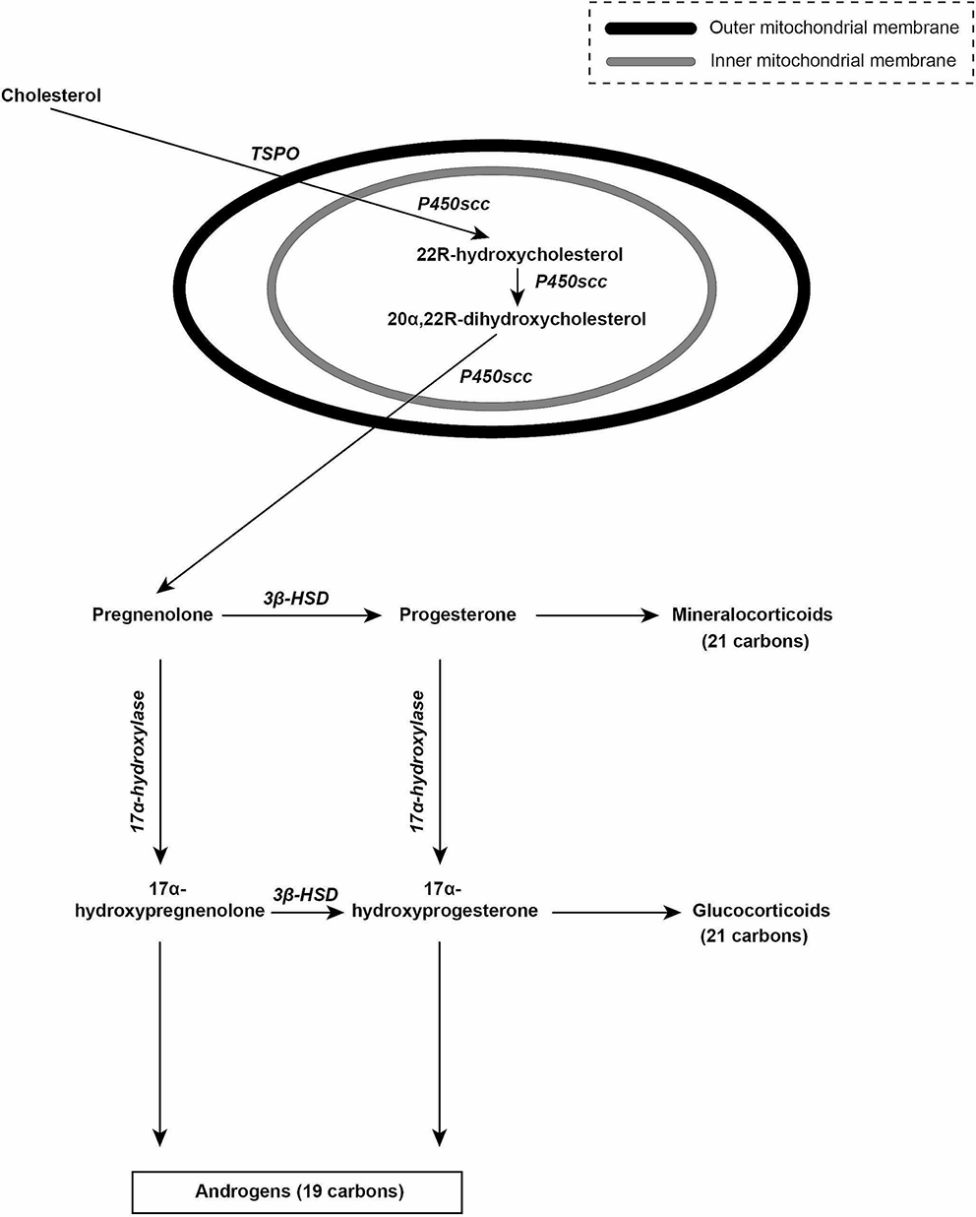
Biosynthesis of progesterone in the intra- and extra- mitochondrial cellular compartment and pathways of its metabolism to steroid hormones. translocator protein (TSPO), cholesterol side-chain cleavage enzyme (P450scc), 3β-hydroxysteroid dehydrogenase/Δ^5−4^-isomerase (3β-HSD)

*Progesterone* is a C21-steroid hormone (*pregn-4-ene-3,20-dione*) [Fig. 1] which binds to plasma proteins such as *albumin* (over 80%, with low affinity; 42 min half-life) and *corticosteroid binding globulin* or *transcortin* (approximately 15–20%, with high affinity; 6 min half-life) [2]. The biological half-life of *unbound progesterone* in circulation is very short (approximately 5 min) [3]. Following intravenous injection, its half-life ranges widely from 3 to 90 min. Thus, although many authors suggest a single daily administation of *MP* per os, it is wiser to administrate it in two daily doses separated by 12 h. This way of administration might represent an inconvenience. Its metabolism is almost completed in first-pass through the liver and other organs (i.e. adrenal gland, ovary, gastrointestinal mucosa, brain, skin) [2]. *Progesterone* is synthesized by *cholesterol* (in two main steps) in the adrenal gland, the ovaries, the testis, the placenta and the central nervous system (CNS). *Cholesterol* undergoes double oxidation to two intermediate-diols (*22R-hydroxycholesterol, 20α,22R-dihydroxycholesterol*) [Fig. 1]. The latter is further oxidized [*via cholesterol side-chain cleavage enzyme* at the inner mitochondrial membrane] to *pregnenolone*, which is further converted to *progesterone* [4] [Fig. 1]. *Progesterone* is metabolized to *17α-hydroxyprogesterone*. In the liver, *progesterone* can be hydroxylated by not steroid-specific *cytochrome P450* enzymes. Of note, environmental chemicals and some drugs increase *progesterone* hydroxylation, resulting in a decrease in the concentration of *progesterone* in the body [5].

**Fig. 2:**
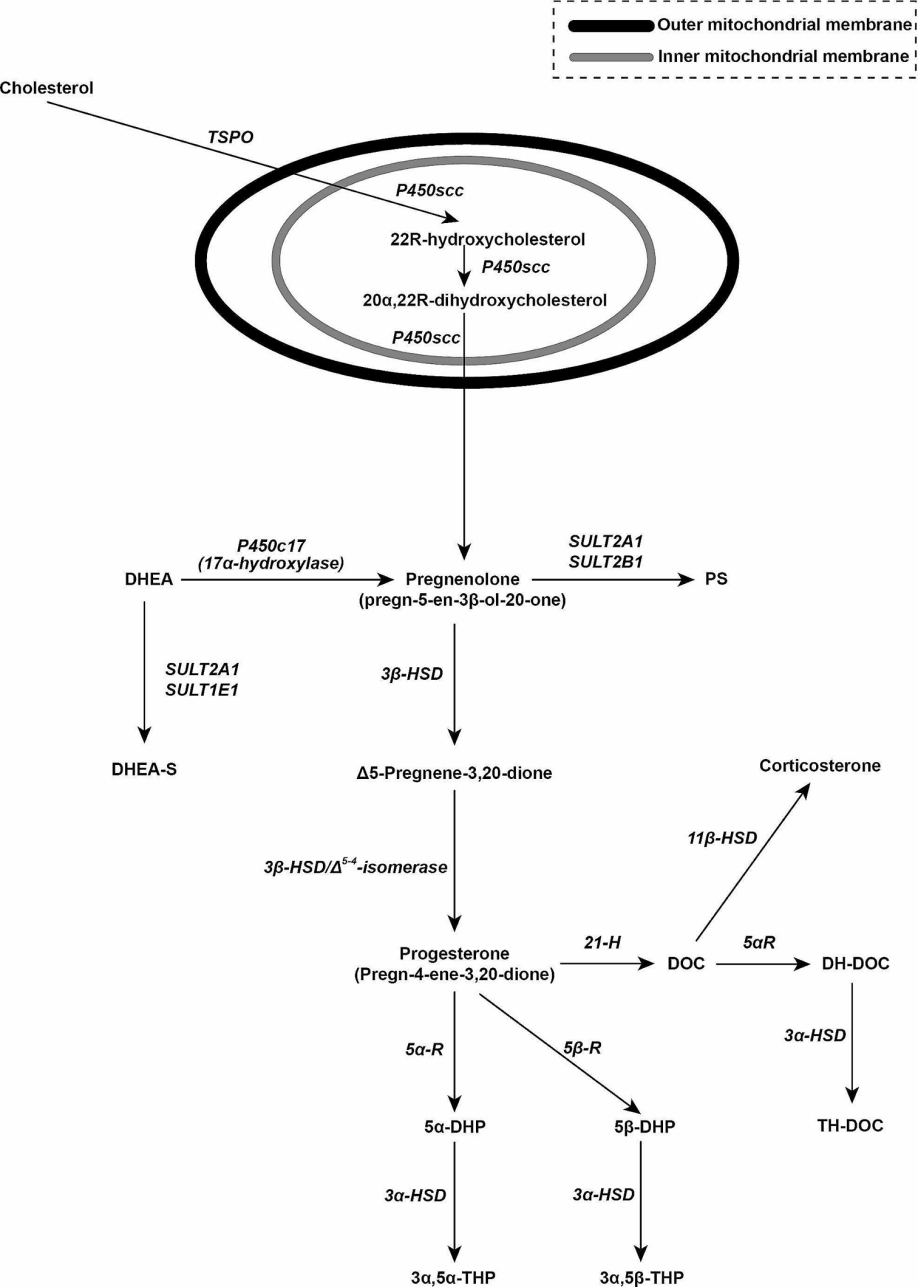
Biosynthesis of progesterone in the intra- and extra- mitochondrial cellular compartment and pathways of its metabolism to neurosteroids. *Prοneurosteroids* [*pregnenolone*, *Δ5-Pregnene-3,20-dione*, *progesterone*, *pregnenolone sulfate* (PS), *deoxycorticosterone* (DOC), *corticosterone*], *pregnane neurosteroids* [*5α- dihydroprogesterone* (5α-DHP), *3α-5α-tetrahydroprogesterone* (3α-5α*-*THP), *5β- dihydroprogesterone* (5β-DHP), *3α-5β-tetrahydroprogesterone* (3α-5β-THP), *dihydrodeoxycorticosterone* (DH-DOC), *tetrahydrodeoxycorticosterone* (TH-DOC)], *androstane neurosteroids* [*dehydroepiandrosterone* (DHEA), *dehydroepiandrosterone-sulfate* (DHEA-S)], *translocator protein* (TSPO), *cholesterol side-chain cleavage enzyme* (P450scc), *DHEA*, *17α-hydroxylase* (P450c17), *sulfotransferase 2 A1* (SULT2A1), *sulfotranferase 2 B1* (SULT2B1), PS, *sulfotranferase 1 E1* (SULT1E1), DHEA-S, *3β-hydroxysteroid dehydrogonase* (3β-HSD), *11β-hydroxysteroid dehydrogonase* (11β-HSD), *21 hydroxylase* (21 H), *deoxycorticosterone* or *11-deoxycorticosterone* or *21-hydroxyprogesterone* (DOC), *5α-reductase* (5α-R), *5α-dihydrodeoxycorticosterone* (DH-DOC), *5β-reductase* (5β-R), *3α-hydroxysteroid dehydrogonase* (3α-HSD), *5α-dihydroprogesterone* or *allopregnanedione* (5α-DHP), *5β-dihydroprogesterone* or *pregnanedione* (5β-DHP), *tetrahydrodeoxycorticosterone* (TH-DOC), *3α-5α-tetrahydroprogesterone* or *allopregnanolone* (3α-5α-THP), *3α-5β-tetrahydroprogesterone* or *pregnanolone* (3α-5β-THP)

*Neurosteroids (neuroactive steroids)* are synthesized either in the brain or in an endocrine gland and then they reach the brain [6]. They are classified in *prοneurosteroids* [*pregnenolone*, *Δ5-pregnene-3,20-dione*, *progesterone*, *pregnenolone sulfate*, *deoxycorticosterone* (*DOC*), *corticosterone*], *pregnane neurosteroids* [*5α-dihydroprogesterone*, *3α,5α-tetrahydroprogesterone*, *5β- dihydroprogesterone*, *3α-5β-tetrahydroprogesterone*, *5α-dihydrodeoxycorticosterone*, *tetrahydrodeoxycorticosterone*], and *androstane neurosteroids* [(*dehydroepiandrosterone* (*DHEA*), *dehydroepiandrosterone- sulfate* (*DHEA-S*)] [Fig. 2]. *Progesterone*, a neurosteroid itself, is synthesized in the central (glia, neurons) and peripheral nervous system [7]. The crucial *cholesterol side-chain cleavage* enzyme is expressed in several brain areas (glia, neurons) in humans, with its greatest concentrations found in the olfactory bulb, caudate nucleus, thalamus, corpus collosum, amygdala, hippocampus, cerebral cortex and cerebellum [8]. In the brain, *pregnenolone* can be converted: to *pregnenolone sulfate*; to *DHEA* in neurons and astrocytes and then *DHEA* can be transformed to *DHEA-S* (*via sulfotransferases*, a conversion not yet clearly demonstrated in the human brain) [9]; to *progesterone* in two steps (*via 3β-hydroxysteroid dehydrogenase* and *3β-hydroxysteroid dehydrogenase/Δ*^*5−4*^*-isomerase activities*, expressed in human oligodendroglial, astrocytes and neuronal cell lines) either in the cytoplasm or in the mitochondria [Fig. 2] [8]. *Progesterone*, in its turn, can be sequentially converted to neuroactive metabolites, such as *5α-dihydroprogesterone* [*via 5α-reductase* (*type 1*, the most abundant isoform expressed in the brain of humans and other species)] and then to *tetrahydroprogesterone* or *allopregnanolone* synthesized *de novo* in the brain (*via 3α- hydroxysteroid dehydrogenase*), or *5β-dihydroprogesterone* [*via 5β-reductase* (the expression of this enzyme in the human brain is debated)] and subsequently to *3α,5β- tetrahydroprogesterone* or *pregnanolone. Progesterone* may also be converted to *DOC* [*via 21-hydroxylase*, the expression of this enzyme in the human brain is debated)] [10]. Subsequently, *DOC* is reduced in the neurosteroids *dihydrodeoxycorticosterone* and *tetrahydrodeoxycorticosterone*. Moreover, *DOC* may alternatively be converted to *corticosterone* [11]. Other neurosteroids which also have clinical and therapeutic importance, such as *dihydrotestosterone* and *androstenedione (A4)*, result *via* the activity of enzymes investigated for their neural activity [9]. Other reductions of *progesterone*, by *5α-* and *5β- reductase* and *3β-hydroxysteroid dehydrogenase*, result in the formation of other neurosteroids such as *iso-* and *epi- pregnanolone*, which, in CNS, are reduced to *pregnanediols* by *20α-* and *20β- hydroxysteroid dehydrogenase.*

### Physiology of progesterone

#### Actions of progesterone through classic progesterone and other receptors

**Fig. 3:**
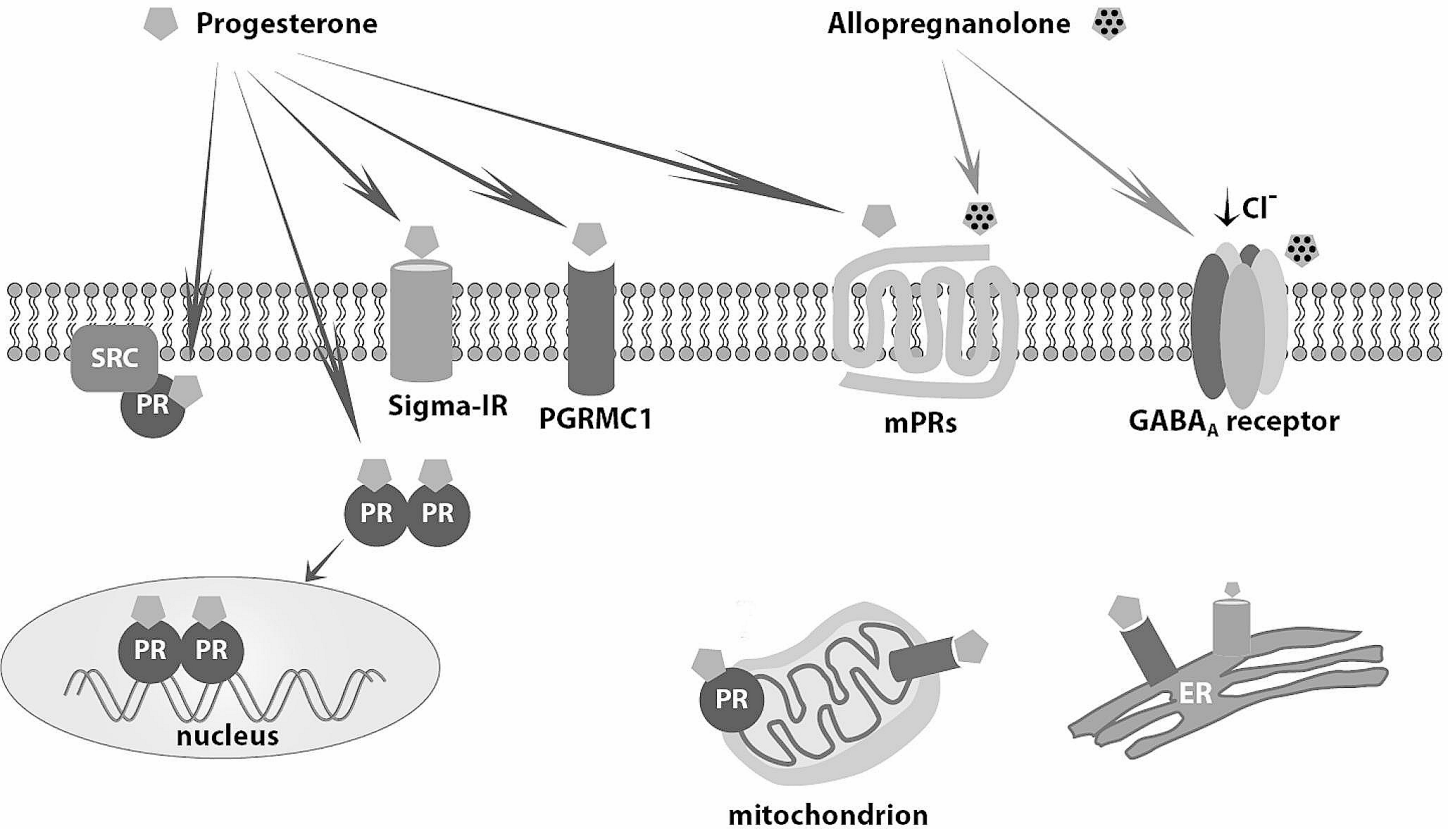
Mediation of progesterone and allopregnanolone cellular actions *via* their membrane, receptors in the cytoplasmic membrane, the nucleus and organelles [*mitochondrion*, *endoplasmic reticulum* (*ER*)] in the central nervous system. *SRC proto-oncogene* (*SRC*), *non-receptor tyrosine kinase* [previously named: *v-src avian sarcoma (Schmidt-Ruppin A-2) viral oncogene homolog*], *progesterone receptor* [(*PR*), the classic nuclear receptor], *sigma-1 receptor* (*Sigma − 1R*), *membrane-associated protein progesterone receptor-membrane component 1* (*PGRMC1*), *progesterone membrane receptor* (*mPR*), *γ-aminobutyric acid receptor A* (*GABA*)_A_

*Progesterone*, secreted from the corpus luteum, dominates the luteal phase of menstrual cycle. *Progesterone* and *progestins* bind to *progesterone receptors* (*PRs)*, while they can interfere with the receptors of other steroid hormones as well [Fig. 3]. *Progesterone receptors* are encoded by a single gene (11q22), are found in the cytoplasm and the nucleus, and are located throughout the body (i.e. in uterus, ovary, vagina, testes, breast, brain, vascular endothelium, thymus, pancreatic islets, osteoblast-like cells, lungs). Their main isoforms are *PR-A* (the commonest) and *PR-B*. The latter seems to act as a positive regulator of *progesterone* on *progesterone response element* (*PRE*). *Progesterone receptor A* (as well as *PR-C*) acts as a transcriptional repressor on *PREs* suppressing the transcriptional activity of *PR-B* as well as those of *estrogen receptors (ER)*, *glucocorticoid*, *androgen* and *mineralocorticoid receptors* [12]. Increased concentrations of *progesterone* during luteal phase as well as *progestins* suppress the expression of *ERα* and *ERβ* in the endometrial epithelium [13]. *Progesterone* and *progestins* inactivate (*via* paracrine mechanisms)*estradiol* (*E*_*2*_) through *17β-hydroxysteroid dehydrogenase type 2* [converts *E*_*2*_ to *estrone* (*E*_*1*_)] and *E*_*1*_*-sulfotransferase* (conjugates *E*_*1*_ to *sulphates*) activities [2]. In addition, *E2* induces the expression of *PRs* mainly *via* its activity on *ER-α* [14]. *Estrogens* differentially regulate *PR-A* and *PR-B* expression in the female hypothalamus, while in the endometrium they increase *PR* synthesis, preparing thus, the endometrium for the secretory phase [15]. Interestingly, in a mouse model, *17β-E2* was shown to regulate the differential expression of the *PR* isoforms in the ventromedial and arcuate nuclei and the medial preoptic area in the hypothalamus where it induced more *PR-A* than *PR-B* [16]. Administration of *progesterone* or *progestins* leads to decrease of *estrogen* and *PRs*. Stromal decidualization and subsequent thinning of the endometrium ensue.

Five isoforms (*α, β, γ, δ, ε*) of *PRs* have also been found on the cell membrane. When bound to *progesterone*, these trans-membrane proteins activate *G-proteins* mediating thus, acute non-genomic actions of *progesterone*. These highly expressed in the brain *PRs* mediate *progesterone* actions upon *gonadotropin releasing hormone* (*GnRH*) secretion, oocyte meiotic maturation, and granulosa cell apoptosis as well as the neuroprotective (anti-apoptotic) effects of *progesterone* and *allopregnanolone* [17]. *Progesterone* is also a ligand of *progesterone receptor membrane component 1*, which appears to mediate non-genomic actions of *progesterone* such as the anti-apoptotic action on granulosa cells [Fig. 3] [18]. *Progesterone* also acts as an antagonist of the *sigma*-*receptors*, both the *sigma 1* and *sigma 2 subtypes*. *Sigma1-receptors* are chaperone proteins at the endoplasmic reticulum; they modulate intracellular calcium mobilization, extracellular calcium influx, *N-methyl-D-aspartate*-mediated actions, *acetylocholine* release, and may interfere with memory, learning, depression, stress reaction, and neuroprotection [Fig. 3] [19]. Furthermore, *progesterone* and some of its metabolites, such as *5β- dihydroprogesterone*, are agonists of the *pregnane X nuclear receptor*, one of the most important defense mechanisms of the human body against toxic substances and chemical insults [20].

*Progesterone* is a potent antagonist of the *mineralocorticoid receptor*; a partial agonist of the *glucocorticoid receptor* (GR); a negative allosteric modulator of *nicotinic acetylcholine receptors*; a positive allosteric modulator of the *γ-amino-butyric acid (GABA)*_*A*_*receptor* acting indirectly *via* its neurosteroid metabolites (such as *5α- dihydroprogesterone* and *allopregnanolone*) [21–22]. Through the *GABA*_*A*_*receptor* it exerts neuroprotective effects and plays a modulatory role in sleep, memory, sexual behavior, respiratory function, control of appetite, weight gain, and tumorigenesis in CNS [22].

#### Progesterone actions in the endocrine and the central nervous systems

*Progesterone* actions in the endocrine system comprise:

In the female reproductive system, *progesterone*: contributes to the control of *luteinizing hormone* (*LH*) and *follicle-stimulating hormone* (*FSH*) secretion and their pulsatility, *via* its receptors on the *kisspeptin*, *neurokinin B*, and *dynorphin* neurons in the hypothalamus under the main control of hypothalamus [23]; controls cyclical proliferation and decidualization of the endometrium; exerts anti-mitogenic actions on endometrial epithelial cells, by counter-acting *estrogen* effects and by modulating *growth factor* pathways [24]; increases the amounts of *insulin growth factor* (IGF)*-binding protein 1* which inactivates *IGF-1* when increased cyclically in endometrial stromal cells; represses *epidermal growth factor*, a mitogenic factor; suppresses the expression of *matrix metalloproteinases* in the same cells; regulates normal menstrual bleeding with *progesterone*-withdrawal bleeding mechanism (*progesterone* or *progestins* lead to cell differentiation, inhibit growth and are considered an inhibitor of endometrial cancerogenesis) [25–26]; enables successful implantation of the ovum; contributes to pregnancy maintenance; decreases maternal immune response and contractility of uterine smooth muscles; participates in the onset of labor. *Progesterone* or *progestins* are produce a secretory endometrium and preserve an established pregnancy. Interestingly, *medroxyprogesterone*, a *progestin*, sits on the osteoblast *PR* and on the breast *glucocorticoid receptor 1* and it is biochemically the closest *progestin* to *MP* [27, 28].

In other endocrine systems, *progesterone*: contributes to the development of the lobuloalveolar breast; controls lactation [29]; peaks in the serum during luteal phase coinciding with increased proliferation of epithelial mammary cells and increased apoptosis (*progesterone* metabolites *3α-* and *20α- hydroxyprogesterone* act as apoptosis-increasing factors) [30]; inhibits competitively *mineralocorticoid receptor* inducing thus, natriuresis, which together with *progesterone*-induced vasodilation may lead to compensatory activation of the *renin-angiotensin-aldosterone* axis [31]; mediates signaling of *insulin* release in pancreatic cells; promotes *gluconeogenesis*, *ketogenesis*, *lipoprotein-lipase* action, fat deposition and protein catabolism [32]; increases core temperature during ovulation; enhances elasticity of the skin (*PRs* are found in the skin) [33]. *Progesterone* is produced and released in high levels by the corpus luteum following ovulation (at least at 5 ng/ml if a successful ovulation happened, while its average concentration is of 2–25 ng/ml). Thus, measuring *progesterone* concentrations or direct *progesterone* effects (i.e. quantitatively documented and sustained increase in core/basal temperature) is the only way in most situations to know that ovulation occurred [34].

In the brain, *progesterone*: increases serotoninergic neurotransmission *via* regulation of *serotonin*-related genes; supports normal brain development; exerts anti-inflammatorry action in damaged brain tissue *via* inhibition of neuron *apoptosis, via* reduction of brain edema and increase of macrophages and microglia near injured tissue, *via* triggering increase of the circulation of endothelial progenitor brain cells and possibly *via* remyelinization in CNS and peripheral nervous system [35–36]. *Progesterone* and *neurosteroids* in CNS act on brain function *via* ligand-gated ion channels and other cell surface receptors. Thus, they impact neuronal excitability by either genomic or rapid, non-genomic ways [6]. *Neurosteroids* (particularly *progesterone* and its metabolites) act beneficially on premenstrual syndrome (PMS), epilepsy, anxiety and depression, learning and memory, alcohol withdrawal, on which they can play a new therapeutic role.

## Pharmacology and physiology of micronized progesterone

*Micronization* is a process which decreases particle size to less than 10 μm, increases the surface area of the active chemical particles and enriches the dilution rate of the micronized compound, increasing thus, gastrointestinal absorption, and bioavailability of the drug. *Progesterone* is lipophilic, stimulates bile salts secretion and *emulsification*, making this way the water-soluble micelles easily absorbed by the lymphatic system and bypassing thus, the first-pass metabolism *via* the liver. Sustained release *MP* tablets were designed by employing hydrophilic polymers which gradually swell, dissolve and erode in a continuous way in order to release the drug in a controlled manner over 16–24 h [37]. With sustained release tablets, there is minimal drug release in gastric fluids while gradual erosion of the polymeric matrix is achieved within the intestine. Thus *MP* escapes *5α-reductase*, a *progesterone*-metabolizing enzyme, found in the upper intestinal wall and the intestinal bacteria. Sustained release *MP* is commercially available since 1986 [37]. The *MP* physiology is almost identical to that of natural *progesterone* demonstrating thus, many of the positive effects of the latter. Several routes of *MP* administration and their respective pharmakocinetic profiles have been tested [38]:

*Per os* administration: Radioimmunoassay measurement of *progesterone*, following *per os* administration of *MP* presents increased cross-reaction with *progesterone* metabolites resulting falsely to 8-fold greater concentrations than those obtained with *liquid chromatography-mass spectrometry* [39]. Mean plasma *progesterone* concentration achieved after *per os* administration of 100 or more mg *MP*, is at least as high as the luteal phase concentrations of *progesterone* [40]. Following 100–300 mg *MP per os*, maximum concentrations of *progesterone* are reached within 2–3 h, remain significantly elevated for up to 12 h and greater than baseline concentrations for at least 24 h. With a *MP* two-dose *per os* regimen (100 mg in the morning and 200 mg in the evening) for 5 days, *progesterone* plasma concentrations remain significantly elevated for up to 36 h, without returning to baseline until after 84 h [41]. A moderate increase in plasma concentration of *progesterone* is related to a greater increase at tissue level (i.e. endometrium, myometrium, breast, glandular and adipose tissue) remaining in therapeutic range of concentrations throughout 24 h [42]. The absorption of *MP per os* is related to individual differences regarding the site of absorption in the gastrointestinal tract and the level of absorption in adipose tissue [1]. Food ingestion enhances absorption of *MP* without altering the time of peak concentration [38]. It seems that maximum achieved concentration of *MP* augments with age, as a result of increased absorption and decreased clearance [38–42]. Although oral *MP* has been associated with more frequent drowsiness and dizziness in some patients, it can be well tolerated with nocturnal administration [43]. With oral administration, *progesterone* accounts for less than 20% of the dose in circulation while 5α- and 5β- reduced products, such as *allopregnanolone* and *pregnanolone*, account for around 80%. *Micronized progesterone* is less effective when administrated *per os* than *via* vaginal or muscular administration. With vaginal administration, circulating *progesterone* accounts for around 50% of the dose and its *5α-* and *5β-* reduced metabolites account for around 40% [44]. When *MP* is administrated *per os*, circulating *DOC* concentrations increase during the first hour after the oral administration and arrive at the maximum concentrations four hours later. *Progesterone* conversion to *DOC* occurs in the duodenum. Circulating *DOC* concentrations increase also following injectable *MP* forms in a more stable pattern. Increased *DOC* concentrations could reduce the antialdosteronic and anti-inflammatory effect of oral *progesterone* more than in the case of vaginal or parenteral administration [44].

Intravaginal administration: Intravaginal *MP* administration results to normal secretory transformation of the endometrium, possibly due to a first-pass *progesterone* uterine effect, while when compared to *per os MP* administration, it demonstrates a slower drug metabolism, longer time to peak concentration, and more constant, plateau-profile, plasma *progesterone* concentrations over time [45–46].

Intramuscular administration: It induces significantly greater peak plasma concentrations and areas under the concentration curve as compared to *per os MP* administration. Peak concentrations are achieved 8 h after administration, later than with *per os MP*, and decline thereafter, but still remain within the luteal phase range after 48 h [44].

Apart the soft *MP* capsules, hard *MP* capsules were introduced in drug industry, in 2004. This *progesterone* capsule is synthesized by *diosgenin* from the plant *Dioscorea zingiberensis* resulting into a chemical structure similar to that of natural *progesterone*. It is produced by solid dispersion at the nanometer level, in order to enhance *progesterone* dissolution rate. In a prospective randomized open-label trial from China, hard *versus* soft *MP* capsules were compared when administrated either *per os* or vaginally. In *per os* administration, metabolism and absorption of soft *MP* capsules were superior to that of hard capsules, while in vaginal administration hard *MP* capsules produce greater absorption concentrations and better pharmacokinetic profile [47].

## Use of oral micronized progesterone in endocrinology

During reproductive life, *MP* is used for diagnostic purposes in the evaluation of primary or secondary amenorrhea. In addition, the use of *MP*, alone or combined with *estrogens* is a useful therapeutic tool in various endocrine disorders during the reproductive and menopausal life of women [Table 1].

**Table 1:** Use of oral micronized progesterone in endocrine practice

Diagnostic use (progesterone or progestin challenge test)
Primary and secondary amenorrhea
Therapeutic use
Primary amenorrhea
Abnormal uterine bleeding associated to ovulatory dysfunction
Luteal phase deficiency
Polycystic ovary syndrome
Secondary amenorrhea
Functional hypothalamic amenorrhea
Premature ovarian insufficiency
Perimenopause
Menopause

### Diagnostic use of micronized progesterone

The administration of a *progesterone* or *progestins* in a challenge test can provide information about a patient’s adequate endometrial estrogenization and outflow tract integrity and the *hypothalamic-pituitary-ovarian* (*HPO*) axis functionality. Because the use of *MP* is safe during pregnancy, it can be prescribed without hesitation as a challenge test [48]. In the litterature, *MP* has been prescribed *per os* in variable regimens: 300 mg once per day for 10 days [39]; 100 mg in the morning and 200 mg before bedtime for 7 days [49]; 400 mg once per day for 7 to 10 days [50]; 200 mg per day for 7 days [51]; 300 mg per day for 5–7 days [52]; 200 to 300 mg per day for 10 days [53]. It appears that there is no established consensus protocol for the administration of *MP* in this test. When 300 or 200 mg *MP vs. placebo* were administrated daily *per os* for 10 days in women 18 to 52 years old suffering from secondary amenorrhea or oligomenorrhea, withdrawal bleeding occurred in 90% of women on 300 mg, in 58% of women on 200 mg, and in 29% of women on *placebo* [48]. Side effects did not differ significantly among the studied groups. However, because some patients do not tolerate well *progesterone*, a shorter (5-day) course of 200 or 300 mg per day of *MP* is proposed. A *progestin* is often administrated for much shorter periods to induce menstruation and may be more appropriate in patients who do not tolerate well *MP*. In absence of withdrawal bleeding, this test can be repeated a few weeks later for 10 days with greater dose (300–400 mg *MP* per day). The test is considered positive when bleeding occurs within 7 days after completion of *progesterone* or *progestin* administration [54]. Absence of withdrawal bleeding may indicate outflow tract obstruction or low endometrial estrogen exposure [54]. The response to *progesterone* or *progestin* challenge can provide additional information about a patient’s estrogen status, especially in those cases in which there is overlay between functional hypothalamic amenorrhea (FHA) and polycystic ovary syndrome (PCOS) [53]. Moreover, *progesterone* or *progestin* challenge test can be prescribed to women presenting with amenorrhea or oligomenorrhea (particularly in PCOS) for induction of withdrawal bleeding in order to time blood-sampling for diagnostic purposes (hormonal measurements) in early follicular phase.

### Therapeutic use of micronized progesterone

#### Primary amenorrhea

Primary amenorrhea is the failure of initiation of menses by the age of 13 in absence of secondary sexual characteristics or absence of menarche by the age of 15 regardless of the presence of normal growth and development of secondary sexual characteristics [55]. Its etiology comprises genetic, anatomical and functional defects including gonadal dysgenesis (i.e. Turner syndrome) (43%), müllerian agenesis (15%), physiological delay of puberty (14%), PCOS (7%), isolated *GnRH* deficiency (5%), transverse vaginal septum (3%), hypothalamic amenorrhea (due to stress and/or energy deficit) (2%) and hypopituitarism (2%). Less common etiologies (≤ 1% each) include imperforate hymen, complete androgen insensitivity syndrome, hyperprolactinemia/prolactinoma, other pituitary tumors, congenital adrenal hyperplasia, CNS defects, craniopharyngioma, Cushing syndrome and hypothyroidism [56–57]. Τreatment depends on the etiology and aims at correcting the underlying pathology, enabling fertility and preventing complications such as osteoporosis. Patients with uterus and primary (i.e. gonadal dysgenesis, etc.), secondary (i.e. hypopituitarism, etc.) or tertiary (i.e. isolated GnRH deficiency, hypothalamic amenorrhea, etc.) will need initiation of hormone therapy, consisting of *estrogen* only (if they present inadequate breast development) or *estrogen* combined with cyclic *progesterone* or *progestins* for patients with a uterus and fully developed breasts. *Progesterone* or *progestins* administration will restore cycles and reduce the risk for endometrial hyperplasia [57]. *Estrogen* therapy is initiated with low doses between 11 and 12 years of age. After 2 years approximately of *estrogen* treatment, or earlier, particularly if a patient presents menstrual bleeding or spotting, *progesterone* or *progestins* should be added [58]. *Micronized progesterone* appears as a good therapeutic option because of its favorable safety profile, especially with regard to breast cancer and veno-thromboembolism risk, particularly in case of patients who present an increased risk of cardiovascular and all-cause mortality (i.e. Turner syndrome) [58–59]. A dose of 200–300 mg per day in a cyclic combined regimen has been proposed [60]. In a non-controlled retrospective study of a Turner syndrome cohort, the prolonged cyclic (10 days per month) use of either *MP* or *medroxyprogesterone acetate* or *levonorgestrel* showed no metabolic change, except for weight gain, which was significantly lesser with *levonorgestrel* [61]. Regimens of *estrogen* plus *progesterone* or *progestins* are cyclic combining an *estrogen* and *progesterone* or a *progestin* (for 10 to 14 days) [62]. Of note, *MP* plus transdermal *estrogen* treatment has been successfully used in patients with Turner syndrome undergoing in vitro fertilization (IVF) as well [63]. However, use of *progestins* is sometimes more appropriate than *MP* use. This could be the case in primary amenorrhea in Turner syndrome, when the side effect of weight gain is concerned [61]. In a double-blind 1-year randomized controlled trial (RCT) possible prevention of bone density loss was investigated in young women who suffered amenorrhea or ovulatory disturbances. Specifically, 61 physically activated normal-weight premenopausal women, who suffered the full range of hypothalamic adaptive, reversible reproductive suppressive changes, were enrolled in the study [64]. The full range of hypothalamic changes includes: amenorrhea, oligomenorrhea, anovulation, or short luteal phase cycles [65]. The participants were divided into four groups receiving cyclic medroxyprogesterone (10 mg/day for 10 days per month) plus calcium carbonate (1,000 mg/day); cyclic medroxyprogesterone with calcium *placebo* group; medroxyprogesterone *placebo* with calcium carbonate; and medroxyprogesterone *placebo* and calcium *placebo*, respectively. Bone density improved in women under cyclic *medroxyprogesterone acetate*, whereas in the *placebo* group bone density decreased. Calcium supplementation presented positive results but without statistical significance [64].

#### Abnormal uterine bleeding associated to ovulatory dysfunction

A positive therapeutic effect of *progesterone* in abnormal uterine bleeding (AUB) was recognized in the first half of 20th century. According to the International Federation of Gynecology and Obstetrics, chronic AUB is a bleeding from the uterine corpus which is abnormal in duration, volume, frequency, and/or regularity (cycle variation: ±4 days; cycles of ≥ 24 days to ≤ 38 days are considered normal in terms of frequency), and it has been present for the majority of the preceding 6 months. Acute AUB is an episode of heavy bleeding, requiring immediate intervention [66]. The term dysfunctional uterine bleeding (DUB), was previously employed in the absence of structural cause of AUB. Furthermore, ovulatory dysfunction (AUB-O), which may be present in a spectrum of menstrual abnormalities, encompasses disorders in which ovulation is either absent or infrequent or irregular [67]. Irregular *progesterone* production from the corpus luteum is involved in these pathologies [67].

In adolescence: In the first post-menarche year at least 50% of cycles are anovulatory and they can remain anovulatory for some years, propably due to improper function of the positive feedback of *E*_*2*_ to *LH* and subsequent absence of midcycle *LH* surge (immaturity or dysregulation of the *HPO* axis) [68]. Monophasic and anovulatory menstrual cycles ensue, follicles undergo atresia and become cystic, while *progesterone* is not produced because of non-developed corpus luteum [69]. Unopposed activity of *estrogens*, derived from ovarian follicles as well as from extragonadal *androgen* aromatization, induces endometrial proliferation followed by abnormal shedding of the endometrium [69]. Of note, obesity is an important contributor to anovulatory cycles in adolescents, while in some young women, particularly those with high body mass index, AUB may be an early presentation of PCOS [70]. Management of AUB in adolescence aims at preventing anemia and at establishing regular cyclic bleeding [71]. *Progesterone* or *progestins* are efficient in treating AUB due to AUB-O by stabilizing endometrial fragility and triggering apoptosis, thus inhibiting growth of the endometrium, while they inhibit angiogenesis and stimulate conversion of *E*_*2*_ to the less active *E*_*1*_ [71]. There is a great variation in the dose and type of *progesterone* or *progestin* as well as the schedule of administration proposed. *Progestin*-only therapy may be used to mature and slough the endometrium [72]. Oral *MP* can be used alone for maintenance therapy, at a dose of 200 mg each night for the first 12 days of each calendar month [73]. Another regimen of *MP* (200-400 mg, once daily for 2 weeks every 4 weeks) has been proposed for the treatment of heavy menstrual bleeding attributed to AUB-O [73]. The same daily dose of *MP* has been proposed for a longer period (for 21 days, starting on day 5) [73]. When initial control of bleeding is achieved, continuation of a *per os progesterone-* or *progestin*- only cyclic regimen (10–14 days starting on the 16th or 12th day of the cycle, respectively) is suggested for at least 6 months after bleeding control [74]. Cyclic *progesterone* therapy could be also recommended for adolescents with irregular cycles, far-apart cycles or heavy flow. It could be prescribed as oral *MP* therapy of 300 mg per day at bedtime. For adolescent patients with menstrual cycles of usually 26 to 32 days, suggested regimen is cyclic administration of *MP* from 14th to 27th day. Otherwise, in presence of shorter cycles (i.e. 21 to 26 days or shorter), suggested cyclic *MP* regimen could be initiated on the 12th up to the 25th day [75]. Sometimes an unpredictable heavy flow may occur after cyclic *MP* therapy. Clinical experience suggests that if this heavy or early flow persists, then the woman may need either an increase of the dose of cyclic *MP* therapy to 400 mg or daily *MP* treatment for three months [75].

In reproductive age: Among other etiologies, AUB ensues from hormonal disorders associated to AUB-O (chronic estrogen stimulation of the endometrium seen in *PCOS* or in *hypothyroidism*) [66]. Therapy should be tailored to each patient’s needs, taking into account medical history and comorbidities. In an RCT, vaginal *MP* or *per os dydrogesterone* were administrated cyclically to women with DUB for 3 months, resulting to a regular bleeding pattern at the end of the first month in 93% and 81% of the patients, respectively. No statistically significant difference was shown in menstrual recordings and endometrial histology between the two groups at the end of the treatment [76].

#### Luteal phase deficiency

Luteal phase deficiency is a condition of insufficient *progesterone* production to maintain a regular secretory endometrium and to allow normal embryo implantation and growth, although certain authors have disputed the contribution of this pathologic entity to subfertility [77]. It may increase the frequency of iron-deficiency anemia due to repeated cycles shorter than 24 days which are often accompanied by premenstrual spotting [78]. Currently, there is no pathognomonic test for the diagnosis of this pathologic entity. It is encountered in women with polycystic ovarian morphology, PCOS, thyroid and *prolactin* disorders, FHA, obesity, or it could be iatrogenic, due to assisted reproduction-related interventions [77]. To treat luteal phase deficiency, the underlying condition should be identified and corrected. Treatment with *MP* or a *progestin* seems reasonable although substantial studies on this therapeutic approach are missing. There is no consensus for the use of *MP* in luteal phase insufficiency.

#### Premenstrual syndrome

Premenstrual syndrome is characterized by recurrent moderate affective and physical symptoms that occur during the luteal phase in 3–8% of women and resolve with menstruation [79]. Clinically significant PMS consists of at least one affective (i.e. angry outbursts, exhaustion, anxiety, irritability, depression and mood swings) or somatic (i.e. bloating, breast tenderness, joint pain) symptom occurring during the five days before the onset of menses while present in at least three consecutive menstrual cycles [80]. The term premenstrual dysphoric disorder refers to the most severe form of PMS, in which symptoms of anger, irritability and internal tension are prominent [80]. Etiologically, an *estrogen/progesterone* imbalance in favor of the former has been proposed as the underlying basis for this syndrome [81], although patients with PMS and premenstrual dysphoric disorder seem to have normal concentrations of serum *estrogen* and *progesterone* [82]. It has been hypothesized that, in genetically predisposed women, PMS results from alterations in female gonadal hormones and CNS neurotransmitters such as *serotonin* (which appears to play the most important role), *GABA*, *endorphins* and other *neurosteroid modulators* [83]. Certain *progesterone* metabolites are *barbiturate*-like *modulators* of *GABA receptors* [84]. *Allopregnanolone* and *pregnanolone* are propably responsible for the anxiolytic and hypnotic effects of *per os* administrated *progesterone* [84] [Fig. 2]. In women with premenstrual dysphoric disorder, alterations in these *neurosteroid* metabolic pathways result in decreased conversion to either *pregnanolone* and/or *DOC*-derived neurosteroids [85], while patients with PMS present reduced sensitivity for *GABA*_*A*_*receptor* allosteric agonists [86] [Fig. 2]. Luteal phase *progesterone* supplementation for PMS treatment might increase the concentrations of *progesterone* metabolites and, subsequently, enhance *GABA*_*A*_*receptor* activation in the CNS [87]. Following vaginal administration of *progesterone*, these metabolites are not found in clinically significant concentrations [88]. *Progesterone* supplementation decreases luteal water retention due to its diuretic properties (promotion of renal sodium excretion) counteracting thus, fluid retention and bloating symptoms associated with increased activity of the plasma *renin* activity and aldosterone system in PMS [89].

In a 4-month double-blind crossover RCT with administration of *MP per os* or *placebo* to women with PMS, a beneficial effect of *progesterone* treatment for affective symptoms (anxiety, depression and stress) and somatic symptoms (swelling, hot flushes) was observed [90]. On the other hand, in a 3-month double-blind, *placebo*-controlled RCT in women with PMS, luteal administration of *MP per os* in high daily doses (1200 mg initially, 1760 mg mean dosage in the third treatment cycle) was more effective than *placebo* for the somatic symptoms, although no better regarding the overall symptomatology [91]. Furthermore, in a double-blind crossover trial, comparing premenstrual administration of *MP per os* with vaginal *MP* or matched *placebo* for 10 days in women with PMS, no difference was found among the three groups, in spite of a significant positive effect upon affective and somatic symptoms in all intervention groups [92]. Interestingly, in the *per os MP* administration group, increased concentrations of *5α-* and *5β- pregnanolone* in serum were found while a significant negative correlation between *5α-pregnanolone* concentrations and anxiety scores was observed [92]. A meta-analysis of these three trials demonstrated a significant positive effect of *MP per os* over *placebo* [93]. In contrast, when RCTs on administration of *progesterone* suppositories or pessaries were analyzed, *progesterone* had no beneficial effect on PMS symptoms [93]. From the existing data, it seems that treatment with *MP per os* might be beneficial for managing PMS symptoms. Of note, *progesterone* hypersensitivity is sometimes linked to PMS. Sensitivity to sex hormones was initially reported in 1921 by H. Geber, who demonstrated a flare in the cyclic urticaria following administration of the patient’s own pre-menstrual serum [94]. Subsequently, cases of severe generalized erythema multiform caused by sensitivity to *progesterone* have been reported [95]. Perimenstrual fluctuations of sex hormones in women is probably, at least partially, responsible for different perimenstrual symptoms especially in autoimmune and pain-related conditions, such as headache and pelvic pain. Thus, severe PMS is related with high predominance (> 90%) of sensitivity to one’s own female hormones, such as *progesterone* and *estradiol* [95].

#### Polycystic ovary syndrome

Polycystic ovary syndrome represents the most common endocrine-metabolic disorder of reproductive age in women. Its prevalence, depending on different definitions, varies between 5 and 14% [96]. These definitions comprise clinical or biochemical hyperandrogenism, oligoanovulation, and/or polycystic ovarian morphology, while the majority of women with PCOS are insulin resistant and hyperinsulinemic [96–98]. Inadequate production of ovarian *progesterone* concentrations during both early, mid- and late luteal phase has been found in infertile women with either polycystic ovarian morphology or oligomenorrhea, as well as in women with PCOS-associated anovulation [97–100]. Luteal *progesterone* concentrations increase in PCOS women following treatment with metformin, suggesting that hyperinsulinemia/*insulin* resistance may be responsible directly and/or indirectly for the decreased *progesterone* concentrations. An inverse relationship between fasting *insulin* and *progesterone* sensitivity in the hypothalamus has been shown in hyperandrogenemic adolescent girls implying that *insulin* resistance may modulate hypothalamic sensitivity to *progesterone* as well [101]. Decreased luteal *progesterone* production contributes to increased frequency of *GnRH* pulsatiliy which results in increased *LH* production [102]. Increased *LH* concentrations, in their turn, lead to additional excessive ovarian *androgen* production, initiating thus, a vicious circle [101]. Of note, administration of *flutamide*, an *anti-androgen*, restores sensitivity of the *GnRH* pulse generator to the negative feedback of *E*_*2*_ and *progesterone* [101, 103]. In female rats, experimental hyperandrogenemia suppresses *PR* mRNA expression in the preoptic area in the hypothalamus, and abolishes *LH* surges [104]. To override the hyperandrogenemia-induced impairment of *progesterone* regulation of *GnRH* pulsatility and normalize menstrual cycle, treatment with *MP* in the adequate posology could be proposed [100] [Table 2]. There is a paucity of data on the utility and usage of *MP* as therapy for hyperandrogenemia and irregular cycles, in women with PCOS. In these women, short-term treatment with *MP per os* resulted either to isolated reduction of *LH* concentrations alone or together with total *testosterone* concentrations [103] [Table 2]. *Per os MP* had beneficial effects on *insulin* resistance or *lipids* in these women [51, 105].

**Table 2:** Suggested doses, administration routes, regimens and side effects of micronized progesterone administrated in PCOS patients [day (d), once a day (q.d.)] (*Concentrations of 17-OH P and HOMA-IR values increased and decreased, respectively, following MP administration. Progesterone and 17-OH P concentrations were significantly lower during menstrual bleeding, whereas LH concentrations were significantly decreased and HOMA-IR values increased)

Reference	Aim	Regimen and administration route	Side effect	Primary outcome
Woods et al., 2002 [49]	Induction of withdrawal bleeding	100 mg MP q.d. *per os* (morning) and 200 mg MP q.d. *per os* (before bedtime) x 7 d	Non reported	Effects on androgens
Livadas et al., 2010 [51]	Induction of withdrawal bleeding	200 mg MP q.d. *per os* x 7d	Non reported	Effects on hormones and glucose metabolism*
Bagis et al., 2002 [105]	Evaluation of insulin sensitivity	300 mg MP q.d. *per os* or 600 mg MP vaginally x 10d	Non reported	Effects on glucose metabolism, lipids and hormones
Stanosz et al., 2014 [109]	Avoid hyperplastic endometrium in PCOS patients with preinvasive endometrial cancer	50 mg MP q.d. vaginally x 6d followed by 100 mg MP q.d. vaginally x 6d, plus 17β-E_2_ transdermally (22 d cycle)	Non reported	Effects on hormones, lipids and endometrium

In PCOS, endometrial hyperplasia ensues from persistent, due to anovulation, *estrogen* stimulation, while moderately elevated concentrations of *E*_*2*_ result from the peripheral aromatization of *A4* to *E*_*1*_ in the adipose tissue of the frequently overweight PCOS patients. In addition, free *E*_*2*_ is increased in the circulation in the context of hyperinsulinemia, due in part to *insulin* down-regulation of *sex-hormone-binding globulin* (*SHBG*) [106]. In face of this accentuated unopposed *estrogen* activity in women with PCOS, *per os MP* is beneficial, regarding endometrial hyperplasia, while it downregulates endometrial *androgen receptors*, found in increased numbers in these women [107–108] [Table 2]. In young women with PCOS and concomitant, primary, pre-invasive endometrial cancer, a 6-month treatment with vaginally administrated *MP*, in combination with transdermal patches of *E*_*2*,_ led to increased *estrogens*(*E*_*1*_, *E*_*2*_) and *progesterone* concentrations and a normal histopathologic picture without neoplasmatic texture of the endometrium [109] [Table 2]. One year after completion of the study, 14 out of 57 women were pregnant and delivered healthy newborns at term. In PCOS, there are therapeutic choices among *progestins*, such as *spironolactone*. Under certain circumstances, a *progestin* might be administrated for much shorter periods to induce menstruation or to prevent mid-cycle bleeding as it is the case of *spironolactone* therapy in PCOS. Treatment of PCOS with 100 or rarely 200 mg per day of *spironolactone*, for 6 to 9 months, is very effective in most cases of PCOS, for improvement of hirsutism, acne and seborrhea [110].

### Secondary amenorrhea

#### Functional hypothalamic amenorrhea

Functional hypothalamic amenorrhea is a form of chronic anovulation unrelated to identifiable organic causes (diagnosis of exclusion) [53]. Common causes include nutritional restriction and/or excessive exercise, with negative energy balance, and extreme stress, which are associated with functional reduction in *GnRH* drive and subsequent decrease in *LH* pulse frequency [111–112]. While *gonadotropin* concentrations are insufficient to maintain full folliculogenesis and ovulatory ovarian function, the hypothalamic-pituitary-adrenal axis is activated and patients present with relative hypercortisolemia [111–112]. Functional hypothalamic amenorrhea is commonly encountered in female athletes manifesting as the “female athlete triad” (low energy availability, menstrual dysfunction, and low bone mineral density (BMD) [113]. Genetic predisposition and endocrine-disrupting chemicals, such as *bisphenol A* and some *polychlorinated biphenyls*, have been implicated in the pathogenesis of FHA [114–115]. Prolonged follicular or inadequate luteal phases may also be observed. Therapeutically, correction or amelioration of causal behavioral factors can enable ovarian, neuroendocrine, and metabolic recovery. Oral contraceptives do not prevent bone loss in women with FHA [116]. Short-term therapy with transdermal *E*_*2*_ and cyclic *MP* or *progestins per os* have been proposed for adolescents and women without restoration of menses after 6 to 12 months of nutritional, psychological and exercise-related interventions, especially in case of low BMD and/or evidence of skeletal fragility [117]. Suggested regimen includes *17β E*_*2*_ patches (100 µg) applied continuously, in combination with cyclic *MP* 200 mg for 12 days every month, targeting endometrial protection [117]. Hormone therapy, however, and induced menses may give a false sense of security despite persistence of FHA-related deleterious effects if causal factors are not corrected [53].

#### Premature ovarian insufficiency

Premature ovarian insufficiency (POI), previously referred to as premature ovarian failure, manifests as hypergonadotropic hypogonadism before the age of 40 years with poorly understood etiology (no identifiable cause in 90% of the cases) [118]. There is a lack of international consensus on diagnostic criteria for POI. Its incidence is approximately 1 in 250 by age 35 years and 1 in 100 by age 40 years [119]. Patients suffer from climacteric symptoms, subfertility, osteoporosis, and cardiovascular disease [120]. Hormone therapy is recommended til the normal average age of menopause [118, 121]. The European Society of Human Reproduction and Embryology recognizes, in their guideline, strong evidence for *per os* cyclic combined treatment to protect the endomerium [122]. An ACOG Committee on Gynecologic Practice recommended daily *estrogen* therapy with 100 µg of transdermal *E*_*2*_ or 1–2 mg *E*_*2*_*per os*, or 0.625–1.25 mg *conjugated equine estrogens per os*, combined with either cyclic administration of 200 mg of *MP* or 10 mg *medroxyprogesterone acetate per os*, daily, for 12 days each month, or continuous daily administration of 100 mg *MP* or 2.5-5 mg *medroxyprogesterone acetate per os* [123]. Because *MP* appears to have a better safety profile when compared to *progestins* in multiple trials in postmenopausal women, it is considered a first-line therapeutic choice [124]. According to recently published data, both *MP* and *medroxyprogesterone acetate* had a neutral effect on the global coagulation assay, when administrated cyclically in women with POI treated with transdermal *E2* [125]. Although, a positive effect on traditional cardiovascular disease markers was noted only in the *MP* treatment arm, there was no significant change in carotid-femoral pulse wave velocity, a biomarker with greater positive predictive value for cardiovascular events [126]. With regard to treatment tolerability, *MP* appeared to be better tolerated than *medroxyprogesterone acetate* in *estrogen*-treated women with POI, with fewer women reporting psychological concerns (mood swings and irritability) [127]. Compared to a standard regimen with *ethinylestradiol* and *norethisterone per os*, replacement with *E*_*2*_ and vaginal *MP* in women with POI resulted in lower blood pressure, better renal function, and less activation of the *renin-angiotensin* system [128]. Furthermore, in a prospective pilot RCT in patients with POI, administration of vaginal *MP* resulted in significantly more in-phase endometria (examined by endometrial histology) and significantly greater *progesterone* and lower *LH* and *FSH* serum concentrations on day 21 of the cycle (considered as day 7 of the luteal phase) when compared with *dydrogesterone per os* [129]. Vaginal, but not *per os MP* administration led to secretory transformation of the endometrium in POI patients on *estrogen* therapy [130].

#### Perimenopause

The term perimenopause is used to describe the time before and after menopause which begins with the initiation of the early menopausal transition (Stage − 2, according to the Stages of Reproductive Aging Workshop criteria) characterized by a persistent difference of 7 days or more in the length of consecutive cycles, elevated but variable early follicular phase *FSH* concentrations, low *antimüllerian hormone* concentrations and antral follicle count; it ends 12 months after the final menstrual period [131]. Perimenopause-related disorders (i.e. AUB, hot flushes and night sweats) may constitute a significant health burden. Progressive oocyte depletion and abnormal follicular development lead to anovulatory cycles which result to decreased *progesterone* production [68]. Thus, *perimenopause* is characterized by unopposed *estrogen* production [132]. Vasomotor symptoms (VMS), also called hot flushes/flashes and night swets, are the hallmark of the period of perimenopause and menopause. They affect the majority of menopausal women within a range of mild, moderate and severe intensity [133]. They may start during perimenopause or during the postmenopausal period and might last up to 5 to 8 years. The primary reason for treatment of VMS during perimenopause and menopause is the fact that they cause distress in the affected women and a worsering in their quality of life [134]. Vasomotor symptoms have also been associated with adverse effects on other systems, such as the cardiovascular system [135]. Thus, certain authors suggested that the effective treatment of VMS might be useful for treating the effects of menopause upon other areas of female physiology as well [136–137]. Treatment of perimenopausal symptoms is important for the reasons referred above. The hormonal changes during this women’s midlife transition, which is for the majority of the women a long period of 2 to 10 years, include peripheral blood *estradiol* concentrations greater than premenopausal ones and decreased *progesterone* concentrations [131, 138, 139]. In detail, the 2001 Stages of Reproductive Aging Workshop proposed a staging system for ovarian aging including menstrual and qualitative hormonal criteria to define each out of seven stages. In detail, during late reproductive/premenopausal period *FSH* concentrations vary, while *antimüllerian hormone* and *inhibin B* concentrations are low. The last two hormones continue to decrease during all the following perimenopausal and menopausal periods, till very low concentrations found at the early postmenopausal years [131, 138, 139]. In the published literature menopausal hormone therapy seem to be more effective for VMS when compared to *placebo*. However, a 25% of treated women discontinue menopausal hormone therapy due to adverse effects, such as menstrual disturbances, breast pain or lack of effectiveness. Taking this data into consideration, recommendation of menopausal hormone therapy for perimenopausal VMS treatment could not be a universal policy [140]. Regarding oral combined hormonal contraceptive, there is not documented a statistically significant improvement when compared to *placebo* in the treatment of perimenopausal VMS [141]. In addition, according to a pilot 50-days-study there is no statistical difference in the prevalence of VMS in perimenopausal women on *levonorgestrel* intrauterine device (LNG-IUD) with *E2* treatment versus women on *placebo* [142]. There is currently no clear recommendation of how to best and most safely treat perimenopausal VMS [134].

Cyclic *progestin* therapy is proposed to replace the decreased or absent *progesterone* concentrations during the second phase of the cycle at this period of life [68]. Although data are scarce at present, *progesterone* treatment (specifically *MP per os*) may be recommended for different indications in these women (i.e. anemia-inducing heavy menstrual flow due to *estrogen* preponderance over *progesterone* concentrations) [143]. In a RCT in 189 perimenopausal women, comparing treatment with *MP* (300 mg daily *per os*) or *placebo* taken for a 3-month period, self-reported perceived decrease in intensity of VMS and improvement in night sweats were significantly greater in patients treated with *MP vs. placebo* [144]. Participants on daily 300 mg *MP* therapy at bedtime perceived, apart from significantly decreased night sweats and improved sleep quality, significantly decreased perimenopausal interference with daily activities, too. The mean Perimenopausal Interference Questionnaire on the third month of this RCT showed a significantly greater improvement in patients treated with oral *MP* than in those treated with *placebo*. These results suggest women on *MP* therapy have less symptomatic daily lives than those on *placebo* [134]. Duration and intensity of bleeding as well as endometrial thickness decreased significantly, after 6 months of treatment with vaginal application of *MP* for 16 to 12 days from the 10th or 14th day of the menstrual cycle, respectively [145]. In premenopausal women with endometrial hyperplasia and AUB, *MP per os* had significantly fewer metabolic side effects compared to the *progestin lynestrenol* and it was equally effective for endometrial protection, although in women older than 45 years it is less effective than *lynestrenol* [146]. In another trial, 300 mg/d *MP* administrated *per os* on days 15–24 of each cycle for 6 months in women with AUB (related to anovulation or incomplete progesterone effect), although less effective for cycle control compared to 15 mg/d *norethisterone*, it had an apparent advantage over the latter with regard to lipid profile. Many of these patients were perimenopausal. Hyperplastic changes of the endometrium disappeared during the first three cycles of the 6-month therapy with both molecules, but their carry-over effect was short, suggesting that therapy should be administrated long-term to these women [147]. Because perimenopausal women present erratically greater concentrations of *estrogens* and lower *progesterone* concentrations, treatment only with *progesterone* may be advantageous compared to combined hormone therapy [144]. Of note, treatment with *MP* or *dydrogesterone* combined with *E*_*2*_*valerate*, respectively, improved sleep quality of perimenopausal and postmenopausal women who suffered from insomnia in a prospective RCT [148].

While the association between cyclic *progestins* and perimenstrual symptoms, headaches and migraine has long been recognized, there are no data regarding the effect of *MP* on migraine [149]. In a RCT on 172 women (the Perimenopausal Estrogen Replacement Therapy study) *MP* (200 mg/d *per os* for 12 days, every 2 to 3 months) was administrated *per os* for 12 months, for endometrial protection, to euthymic perimenopausal and early postmenopausal women who also received transdermal*E*_*2*_ (patches of 0.1 mg). During the intervention, rates of clinically significant depressive symptoms (CES-D score ≥ 16) were found to be 17% in the active treatment group and 32% in the *placebo* group, suggesting that this combination prevents the development of clinically significant depressive symptoms in this population. This effect was independent from treatment-induced amelioration in menopausal symptoms [150]. In addition, in healthy perimenopausal and early postmenopausal women, aged 45–60, 12-month treatment with 0.1 mg/day transdermal *E*_*2*_ and cyclical 200 mg/day *MP* for 12 days tended to improve cardiac autonomic control and prevented age-related changes in stress reactivity and endothelial function [151].

#### Menopause

Natural menopause is defined as the permanent cessation of menstruation, determined retrospectively, after 12 months of amenorrhea without any other obvious pathologic or physiologic cause [152]. Up to 57% of women report onset or significant increases of VMS at menopause [153], while they admit that hormone therapy is highly effective for the treatment of these symptoms [154]. Because unopposed *estrogens* have been demonstrated to increase the risk for endometrial cancer, a combined *estrogen* and *progestin* continuous hormone therapy is strongly recommended in women with an intact uterus [155]. *Progesterone* and *progestins* do not behave all in a similar way with regard to potential adverse metabolic effects or associated breast cancer risk when combined with long-term *estrogen* therapy [156–158]. Two hundred to 300 mg *MP* daily combined to *estrogen* therapy are devoid of side effects on blood pressure, lipid profile or glucose homeostasis and display physiologic effects on the endometrium [159–162]. This combined therapy with *MP* is a natural and safer option in comparison with combined therapies with *progestins*, though not yet proven in a RCT [163–165].

In a single 3-month randomized double-blind *placebo*-controlled trial in healthy, nonsmoking, menopausal women, 300 mg *MP* alone administrated *per os* daily, were significantly more effective than *placebo* in improving frequency and score of VMS [166] [Table 3]. Four weeks after the end of the trial, the beneficial effect of *MP* (but not with *placebo*) on VMS persisted, although at a lesser extent [167] [Table 3].

**Table 3:** Randomized control trials with administration of MP to post-menopausal women. Suggested doses, administration routes, regimens and side effects of MP and placebo [once a day (q.d.)]. Side effects did not differ between MP and placebo administration, while they were not severe

Reference (participants in MP)	Aim	Regimen and administration route	Side effects of MP	Side effects of placebo	Primary outcome
Hitchcock et al., 2012 [166] (75)	Efficacy of MP regimen	300 mg MP q.d. *per os* at bedtime	Abdominal sensation, depression, anxiety, dizziness, breast lump, daytime drowsiness, dry mouth, facial swelling, rash, vasomotor symptoms, nausea, insomnia, mastalgia, multiday menstrual bleeding.	Insomnia, leg pain, initial daytime drowsiness, weight gain.	Vasomotor symptoms
Prior et al., 2012 [167] (75)	Efficacy of MP regimen	300 mg MP q.d. *per os* at bedtime	No serious side effects	No serious side effects	Vasomotor symptoms, withdrawal effects
Schüssler et al., 2008 [172] (10)	Efficacy of MP regimen	300 mg MP q.d. *per os* in the evening x 21d	Non reported	Non reported	Sleep quality, cognition

When four different *molecules* (*MP, medroxyprogesterone acetate, nomegestrol acetate*, *dydrogesterone*) were administrated for 12 cycles to 100 recently menopaused women for at least 12 months combined with transdermal *E*_*2*_ cyclically, transvaginal ultrasonography showed a similar increase in endometrial thickness after treatment in all groups. Administration of 200 mg/day *MP* was associated with more irregular bleeding pattern and significantly earlier appearing bleeding episodes than in patients receiving the three *progestins* [168]. In another multicenter, randomized, parallel group study, when *chlormadinone acetate* was compared to *MP* combined with transdermal *E*_*2*_ in a combined cyclic therapy in 336 healthy postmenopausal women, *chlormadinone acetate* was as protective regarding endometrial hyperplasia as *MP* (96.3% and 92.0% of success, respectively), but a secretory endometrium was found in 81.5% of the *chlormadinone acetate* patients, compared to only 50.7% in the *MP* group. Unscheduled bleeding, spotting and/or metrorrhagia, were more frequent under *MP* than under *chlormadinone acetate* (17.9% and 13.7%, respectively), while the beneficial effects on hot flushes were more important in the *chlormadinone acetate* than in the *MP* group [169].

In the brain, oral *MP* is rapidly converted into *allopregnanolone*, which, acting *via GABA*_*A*_*receptors*, decreases anxiety and induces sleep [94]. In an unblinded observational study, postmenopausal women who changed their hormone therapy from a *medroxyprogesterone acetate*- to a *MP*- containing regimen experienced improvement in VMS and somatic complaints, as well as in symptoms of anxiety and depression, while fewer side effects and greater overall satisfaction with the *MP*-containing regimen were reported [164]. However, in another 91-day, single-blind pilot randomized study, neither 5 mg/day *medroxyprogesterone acetate* nor 200 mg/day *MP per os*, both combined with *conjugated equine estrogens*, had an effect on mood in normal, non-depressed and non-anxious early postmenopausal women [170]. In any case, menopausal women with intense sleep problems can be effectively treated with oral *MP* alone [167]. The light sedative effect of oral *MP* is created by its metabolites, in particular *5α-* and *5β- allopregnanolone*. *Progestins* are not converted into these metabolites and thus, they have no soporific effect [156]. Administration of oral non-micronized *progesterone* improves falling asleep when given at bedtime and may also restore disturbed sleep [171]. A randomized double blind cross-over trial documented significant increases in early rapid-eye movement sleep, decreased sleep interruption and no changes in morning neurocognitive function in menopausal women treated *per os* with 300 mg *MP* daily compared to *placebo* for 21 days [172] [Table 3]. Interestingly, a recent RCT comparing the sleep quality of peri- and post- menopausal women with insomnia, who received daily 1 mg *E*_*2*_*valerate* combined with either 10mg *dydrogesterone* or 100 mg oral *MP* for 3 months, showed an improvement in sleep quality with both regimens, without significant difference between them [150]. Importantly, a recently published systematic review and meta-analysis of RCTs examining the efficacy of *MP* upon sleep predominantly in postmenopausal women, showed improvement of various sleep parameters on *MP*, including total sleep time and sleep onset latency, and self-reported sleep outcomes, though studies were inconsistent [173]. All studies employing polysomnography showed improvement in sleep parameters with *per os* doses of 200-300mg *MP*. Findings of another recent systematic review and meta-analysis support the use of transdermal *17β-Ε2* combined with *MP* for at least 6 months in menopausal women with sleep disturbance [174].

Regarding memory, in a small double-blind RCT, the administration of 200 mg *MP per os* daily to menopausal women from days 1 to 14 combined with 0.625 mg *conjugated equine estrogens* daily in 28 days cycles resulted to better performance in a working memory test compared to women who received either 10 mg *medroxyprogesterone acetate* or *placebo* combined with 0.625 mg *conjugated equine estrogens* [175]. Thus, it appears that the improvement in working memory was due to *MP* combined with *conjugated equine estrogens* and not *conjugated equine estrogens* alone. Working memory, the ability to hold in mind and manipulate information to produce a response, involves mainly the prefrontal cortex. Thus, the *MP-conjugated equine estrogens* combination could be beneficial on the prefrontal cortex, when its function declines with age [175–176].

## Summary

*Micronized progesterone* is natural *progesterone* presented in a pharmacotechnical structure which increases its bioavailability. As it preserves its full potential of activity, while exempt of many of the side-effects of *progestins*, it may be considered as an attractive diagnostic and therapeutic pharmaceutical substance. For diagnostic purposes, it is used to provide information about a patient’s adequate endometrial estrogenization and outflow tract integrity as well as the HPO axis functionality (*progesterone* or *progestin challenge test*). Therapeutically, oral *MP* is useful in treating several endocrine disorders. In cases of unopposed *estrogen* exposuse, in women with an intact uterus, cyclic or continuous administration of *MP* reduces the risk for endometrial hyperplasia which is associated to AUB and endometrial cancer.

In patients with a uterus and fully developed breasts presenting primary amenorrhea, *estrogen* combined with cyclic *MP* is proposed as hormone therapy. Because of its better safety profile, especially with regard to breast cancer and veno-thromboembolism risk, *MP* is the preferred steroid molecule for individuals with *Turner syndrome*, who exhibit a three- to four- fold increase in cardiovascular and all-cause mortality and an increased risk of metabolic diseases.

In adolescent patients with AUB-O, oral *MP* can be used alone as a regimen for maintenance therapy and for treating heavy menstrual bleeding, while in patients of reproductive age vaginal *MP* has been also shown to be effective [76]. Treatment with *MP* seems a reasonable option for patients with short cycles and anemia in the context of luteal phase insufficiency [78]. Nevertheless, at the moment, there is no consensus regarding *progesterone* administration protocols for this pathologic entity.

Treatment with oral *MP* might be beneficial for managing PMS symptoms, as it has been hypothesized that an *estrogen*/*progesterone* imbalance in favor of the former is implicated in the pathogenesis of PMS [81]. In patients presenting PMS and premenstrual dysphoric disorder, use of *MP per os* during the luteal phase might be justified, in order to increase the concentrations of *progesterone* metabolites and, subsequently, enhance *GABA*_*A*_*receptor* activation in CNS [87]. *Micronized progesterone* decreases luteal water retention due to its diuretic properties counteracting thus, the fluid retention and bloating symptoms of premenstrual dysphoric disorder [89]. Further research is justified regarding the use of *MP* in premenstrual dysphoric disorder due to paucity of RCTs.

In PCOS, treatment with *MP* can be justified by the multiple potential mechanisms of *progesterone* implication in the pathogenesis and manifestations of the syndrome (i.e. inadequate production of ovarian *progesterone* during the luteal phase; decreased hypothalamic sensitivity to *progesterone*-mediated negative feedback due to hyperandrogenemia-induced impairment of *progesterone* regulation of *GnRH* pulsatility) [101–102]. Interestingly, available data are limited. In women with PCOS of reproductive age, treatment with *MP per os* reduces *LH* and total *testosterone* concentrations and exerts beneficial effects on *insulin* resistance and *lipids* [51, 105]. In premenopausal women with PCOS and endometrial hyperplasia, *MP* administrated *per os* controls endometrial hyperplasia [107–108]. Of note, in young women with PCOS and concomitant, primary, preinvasive endometrial cancer, a 6-months vaginal administration of *MP* in combination with transdermal *E*_*2*_ reestablishes normal histopathological endometrial picture [108].

In FHA patients presenting chronic anovulation, cyclic administration of *MP*, combined with transdermal *estrogen* can be beneficial [54]. In principle, therapeutic interventions should, at first, focus on correction or amelioration of the causal nutritional, psychological and exercise-related factors [54, 115]. However, because bone health may be compromised even after 6 to 12 months of amenorrhea, short-term therapy with cyclic administration of *MP*, combined with transdermal *estrogen* (not oral contraceptives, which appear not to prevent bone loss) have been proposed for adolescents and young women [117].

In women with POI, evidence is limited regarding the therapeutic effect and role of *progesterone* or *progestins* used in hormone therapy [118]. In POI, when comparing cyclic to continuous *MP* administration combined with *estrogens*, it appears that cyclic treatment protects better the endometrium [122]. In these patients, *MP* could be considered as first-line therapeutic choice, due to its better safety profile in postmenopausal women, when compared to *progestins* [124].

In perimenopausal women, cyclic *MP per os* has been shown an effective treatment for VMS, peri- and post- menopause-associated insomnia and symptoms of anxiety and depression, while being superior to *norethisterone* with regard to *lipid* profile [147]. Furthermore, MP may be efficacious for anemia-inducing heavy flow, resulting from endometrial hyperplasia due to the imbalance between *E2* and *progesterone* concentrations (in favor of the former) [131–132, 143]. Replacing the decreased or absent *progesterone* concentrations during the second phase of the cycle with chronic oral *MP* administration, in long cycles, stabilizes the endometrium and reduces its thickness. Because these women present erratically greater concentrations of *estrogens*, *progesterone*-only treatment may be advantageous compared to combined hormone therapy [143]. It appears that *MP* seems an efficacious and safe treatment for most perimenopause related disorders.

In menopause, hormone therapy is highly effective for the treatment of VMS [154]. Importantly, *MP* has been shown to be as effective as *progestins* for controlling endometrial growth, with significantly fewer metabolic side effects [158]. *Estrogen* therapy together with 200 to 300 mg *MP per os* daily appear to be devoid of side effects on blood pressure, *lipid* profiles or glucose homeostasis [159–162]. Thus, *MP* use *per os* should be favored in menopausal women, because it is natural, safer and better tolerated compared to *progestins* [158, 161–164]. Though not yet proven in a RCT, combined treatment with oral *MP* and transdermal *E*_*2*_ can be considered as the optimal hormone therapy for menopause [165]. In the brain, oral *MP* is rapidly converted into *allopregnanolone*, which acts through *GABA*_*A*_*receptors* to decrease anxiety and induce sleep [93]. Furthermore, there are findings supporting the association between mood effects and *allopregnanolone* serum concentrations during *progesterone* treatment [175]. The light sedative effect of *MP* is produced by its metabolites, found in clinically significant concentrations only after oral *MP* treatment and not vaginal *MP* administration nor treatment with *progestins* [156]. Importantly, there are data indicating an improvement in working memory in menopausal women treated with a combination of estrogens and *MP* [175].

In summary, *MP* is a natural *steroid hormone* with increased bioavailability exempt of many of the side-effects of *progestins*. Because the use of *MP* is safe during pregnancy, it can be prescribed without hesitation as a *challenge test* [48]. Its favorable safety profile, especially with regard to breast cancer, veno-thromboembolism risk and metabolic profile (*i.e. insulin* resistance, *lipid* profile, blood pressure, renal function), makes *MP* a good therapeutic option particularly in case of patients who present an increased risk of cardiovascular and all-cause mortality (Turner syndrome, menopause) [56, 58, 100, 128]. In several studies in menopausal women, *MP* had been shown to be as effective as *progestins* for controlling endometrial thickness, with significantly fewer metabolic side effects [45, 146]. Furthermore, when administrated *per os*, it acts as a neurosteroid directly or through its metabolites and exerts numerous beneficial effects on brain function.

However, when compared to *progestins*, *MP* was associated to more irregular bleeding in recently menopaused women, but greater doses could probably lead to better cycle control. In *estrogen* treated patients with POI, vaginal, but not *per os MP* administration led to secretory transformation of the endometrium, suggesting reduced bioavailability of *MP* regarding the endometrium upon oral administration. Of note, in a cross-sectional and non-controlled retrospective study of a *Turner syndrome* cohort, the prolonged cyclic use of *MP* was less favorable than *levonorgestrel* with regard to weight gain [61]. Although oral *MP* has been associated with more frequent drowsiness and dizziness in some patients, it can be well tolerated with nocturnal administration [43].

In conclusion, convincing data suggest that *MP* is an excellent therapeutic alternative in several endocrinologic pathologic entities. Tailoring the dose and regimen individually to patients may result in an efficacious treatment with a better safety profile than *progestins*. Further and thorough clinical research, particularly with carefully designed RCTs, should clarify the areas lacking sufficient evidence.

## Abbreviations

A4: androstenedione
ACOG: American College of Obstetricians and Gynecologists
AUB: abnormal uterine bleeding
AUB-O: ovulatory dysfunction
BMD: bone mineral density
CNS: central nervous system
DHEA: dehydroepiandrosterone
DHEA-S: dehydroepiandrosterone- sulfate
DOC: deoxycorticosterone
DUB: dysfunctional uterine bleeding
E2: estradiol
E_1_: estrone
ERs: estrogen receptors
FHA: functional hypothalamic amenorrhea
FSH: follicle-stimulating hormone
GABA: γ-amino-butyric acid
GnRH: gonadotropin releasing hormone
HPO: hypothalamic-pituitary-ovarian
IGF: insulin growth factor
IVF: in vitro fertilization
LH: luteinizing hormone
MP: micronized progesterone
mPR: progesterone membrane receptor
PCOS: polycystic ovary syndrome
PGRMC1: membrane-associated protein progesterone receptor-membrane component 1
PMS: premenstrual syndrome
POI: premature ovarian insufficiency
PR: progesterone receptor
PRE: progesterone response element
RCT: randomized controlled trial
SHBG: sex-hormone-binding globulin
VMS: vasomotor symptoms
17-OHP: 17- OH progesterone
